# Early Physiotherapy as an Adjunct to Surgical Approach in Case of Chronic Tibial Osteomyelitis Treated with Sequestrectomy and an Ilizarov Ring Fixator in a 14-Year-Old Schoolgirl: A Case Report

**DOI:** 10.7759/cureus.29663

**Published:** 2022-09-27

**Authors:** Purva H Mundada, Deepali S Patil

**Affiliations:** 1 Physiotherapy, Ravi Nair Physiotherapy College, Datta Meghe Institute of Medical Sciences (DU), Wardha, IND

**Keywords:** case report, physiotherapy, antibiotic bead, saucerization, sequestrectomy, ilizarov ring fixator, chronic osteomyelitis

## Abstract

Osteomyelitis is an infectious disease of the bone and bone marrow caused by pyogenic organisms that may be acute or chronic. Early diagnosis and treatment prevent complications like limb deformity and limb length discrepancies. A 14-year-old girl presented to the orthopedic department with complaints of pain, fever, swelling, tenderness, and pus discharge across the right leg below the knee. Previously, she visited a local hospital where after initial investigations, she underwent saucerization followed by antibiotic bead application. After experiencing no improvements, she came to Acharya Vinoba Bhave Rural Hospital, Sawangi Meghe, Wardha. After investigations, she was confirmed to have chronic right tibial osteomyelitis, after which she underwent sequestrectomy with an Ilizarov Fixator application. After three months, she started experiencing pain over the right leg, discharge from the wound site, on and off fever, and difficulty walking, for which she was again admitted to this hospital. She was primarily managed by dressing and medication and subsequently received physiotherapy intervention with proper rehabilitation protocol which was found to be very effective in achieving functional mobility and independence. This case study concludes that a multidisciplinary team involving a definitive surgical approach and physiotherapy rehabilitation protocol which resulted in improved functional mobility and independent ambulation for the patient, which plays a significant role in a fast and successful recovery.

## Introduction

Osteomyelitis is a slow, incremental, infectious process affecting the bone cortex and bone marrow that occurs secondary to infection with pyogenic microorganisms [1]. Based on the histopathological observations rather than the length of the infection, osteomyelitis is categorized as acute or chronic. Usually, acute osteomyelitis develops two weeks after bone inflammation and is marked by inflammatory changes in the bone [2]. While, chronic osteomyelitis (COM) sets six or more weeks after bone infection and is better recognized as a long-term bone infection, marked by a tenacious microorganism formation, the appearance of a dead bone with surrounding infected unhealthy granulation tissue, low-grade inflammation, and discharging sinus [2,3]. It is a significant complication of acute osteomyelitis when the latter is not treated promptly and adequately in nearly 10-30% of cases [2,4]. The pathogenesis includes metaphysis infection, followed by the development of a subperiosteal abscess, discharging sinus, and sequestrum (dead bone). The long bones of the lower limb (LL) are commonly involved, the upper end of the tibia and the lower end of the femur are the most common sites, and the humerus from the upper limb (UL) [5]. The most common organism responsible for COM is Staphylococcus aureus (60%), followed by Enterobacteriaceae (23%), Pseudomonas (9%), and Streptococcus (9%). If COM is associated with an implant, S. aureus or S. epidermidis is the typical causative agent [6].

COM may occur as a persistent or sporadic illness. Symptoms and duration of symptoms may differ significantly, while periods of quiescence may also be of variable duration. The rate of relapse is high despite seemingly satisfactory treatment [7]. Clinical characteristics are diverse and usually not unique depending on the patient's age, causative pathogen, region of involvement, and co-morbidities [8]. Recurrent pain, swelling and bone tenderness, limping or muscle spasm with intermittent bouts of low-grade fever, frequently accompanied by recurrent sinus tract with purulent discharge, are the most common clinical symptoms. Cyclical pain that rises in severity in neglected cases is related to fever and subsides when pus breaks out of the sinus [7-9]. Treatment options are sequestrectomy, curettage, saucerization, excision of the sinus tract, and finally amputation in the case of long-standing discharging sinus. In the post-surgery care of patients, physiotherapy is beneficial. "Physiotherapy interventions include passive movements to active assisted movements to active movements, progressive resistive exercises, muscle energy technique, cryotherapy, and electrotherapy for pain alleviation [5,10,11]."

## Case presentation

Patient Description

**Figure 1:**
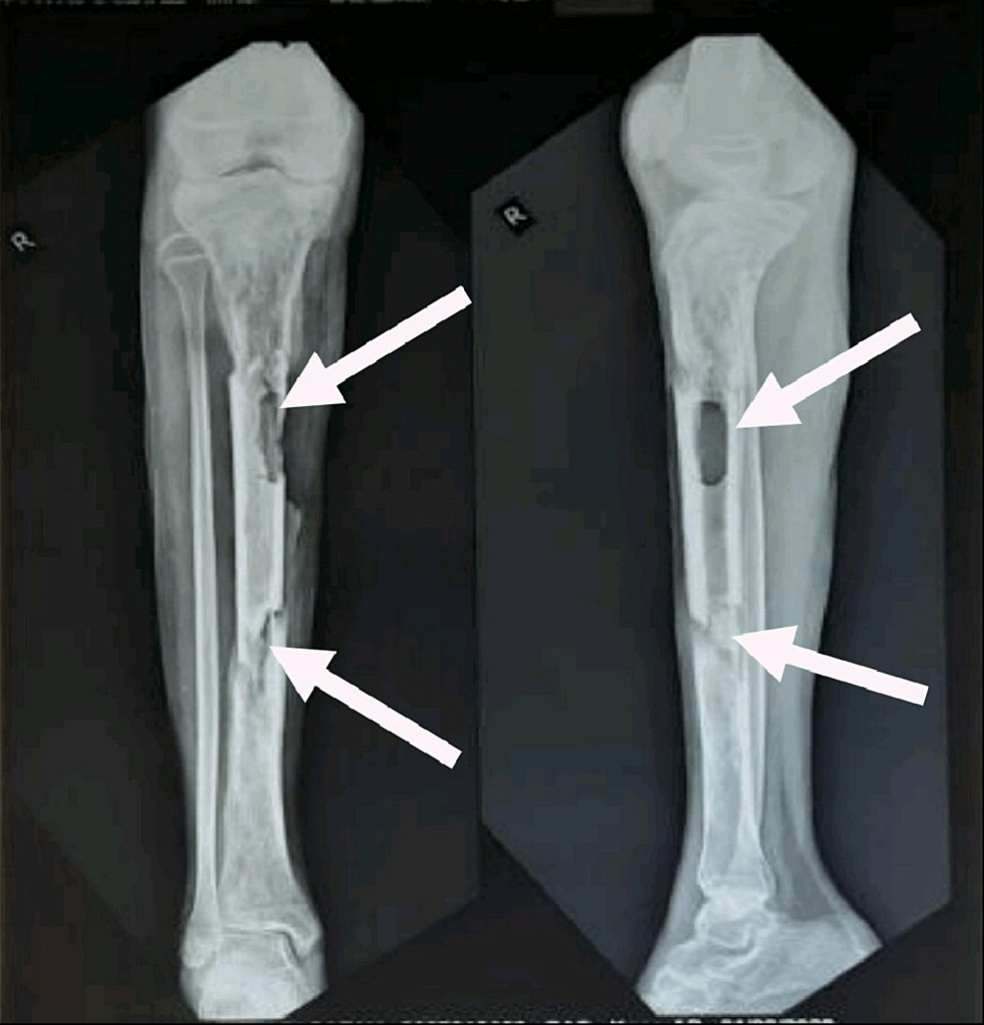
X-ray of the right lower leg (knee AP view) showing diffuse multiple osteolytic lesions with bone defect (sequestrum) mid tibia anteroposterior: AP

**Figure 2:**
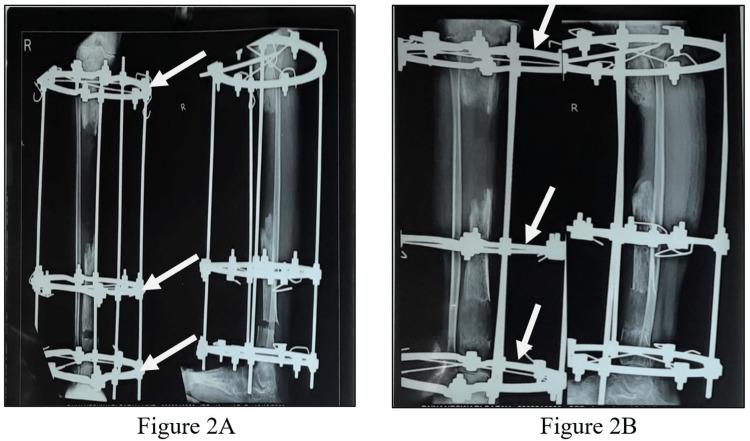
Check X-rays of the right lower leg with Ilizarov ring fixator in situ: A) knee AP view, B) lower leg AP and lateral view anteroposterior: AP

**Figure 3:**
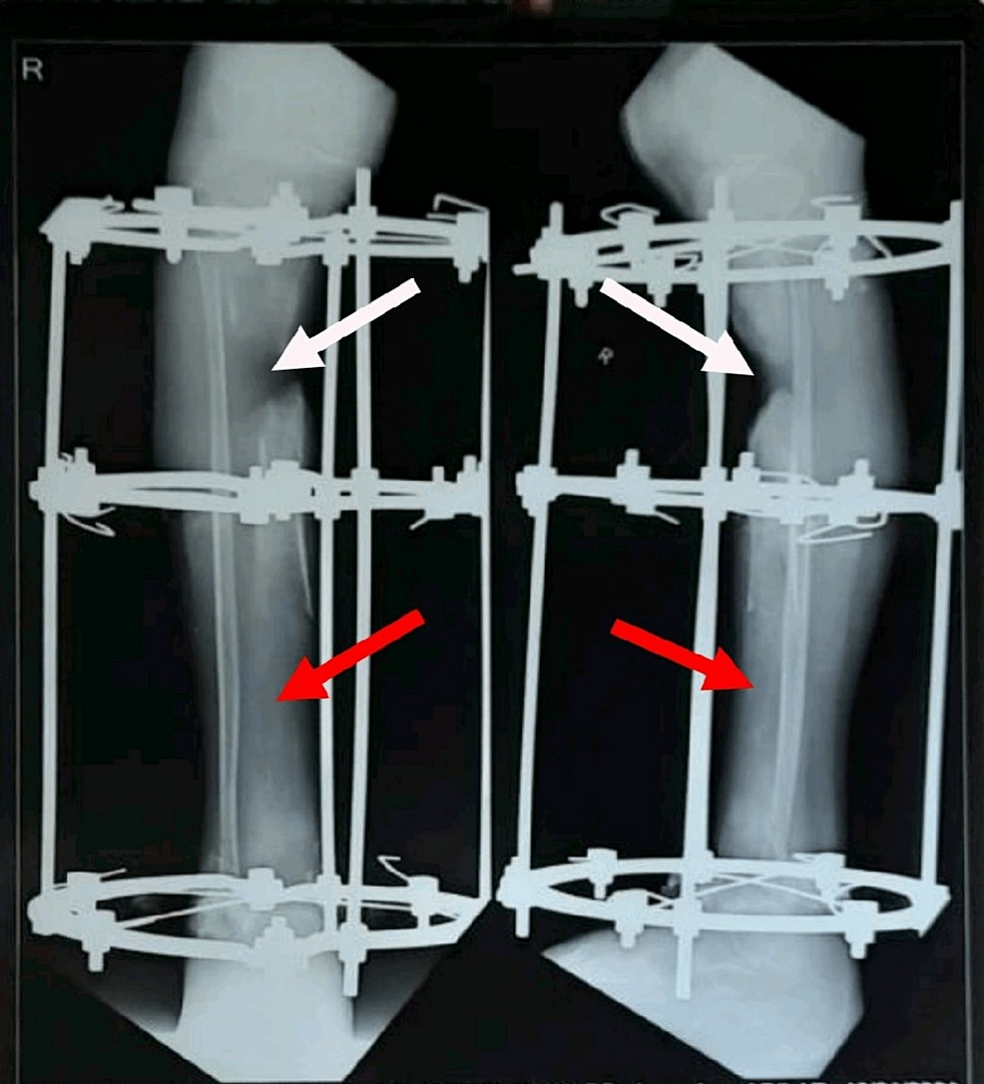
X-ray of the right lower leg (AP and lateral view) with Ilizarov ring in situ showing bone loss of approximately 4cm over the proximal 3rd tibia (white arrows) and new bone formation over the distal 3rd tibia (red arrows) anteroposterior: AP

A patient 14-year-old girl was apparently alright nine months back, then she developed pain, swelling, tenderness, and redness across the right leg below the knee, which was gradually progressive, after a history of falls in May 2020. Later, pus developed in the wound site along with the discharging sinus over the anterior aspect of the right leg. She visited a private hospital in Amravati, where after initial investigations she underwent incision and drainage with saucerization in May 2020. After the surgical procedure, antibiotic bead application was accomplished. Till then, the wound was managed with a regular sterile dressing. Since there was no improvement in her condition, her parents took her to the orthopedic department of Acharya Vinoba Bhave Rural Hospital (AVBRH) in September 2020 with complaints of pain, fever, swelling, tenderness, and pus discharge across the right leg below the knee. An X-ray of the right leg was done, which showed diffuse multiple osteolytic lesions with a bone defect (sequestrum) mid tibia, and thus was diagnosed with chronic right tibial osteomyelitis (Figure 1). She underwent sequestrectomy and Ilizarov fixator application over the right tibia under spinal anesthesia on 29th September. Post-operatively, the wound was managed with a regular sterile dressing. A 0.5 mm distraction was done every 12 hours. Check X-rays were taken every five to seven days till discharge (Figure 2). She was advised to walk with partial weight bearing (PWB). As the patient showed clinical improvement with decreasing discharge from the wound site, she was discharged on October 26th. In December 2020, she started experiencing pain in the right leg and discharge from the wound site. She also complained of an on-and-off fever with difficulty walking, unassociated with any weight loss or night sweats. For the same reason, she was admitted to the orthopedics ward on 12th January 2021. An X-ray of the right leg was performed on the 13th, which interpreted a bone loss of approximately 4 cm over the proximal third tibia. New bone formation was seen over the distal third tibia (Figure 3). She was referred to physiotherapy with complaints of pain in the right LL and difficulty performing movements of the right LL along with difficulty in walking. The pain was insidious in onset, gradually progressive, and dull aching in nature, aggravated by movement and relieved by rest; the pain intensity on a numerical pain rating scale (NPRS) was 5/10 at rest and 8/10 with activity (Table 1).

**Table 1:** NPRS scores pre and post-intervention numerical pain rating scale: NPRS

NPRS	Pre-intervention	Post-intervention
On rest	5/10	0/10
On movement	8/10	2/10

Clinical Findings

**Figure 4:**
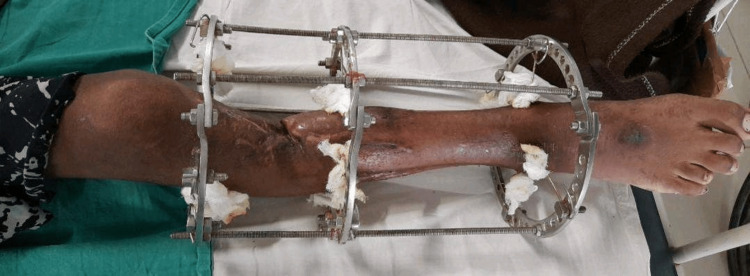
Right lower leg with an Ilizarov ring fixator (3 rings) and visible proximal 3rd of the tibia

After taking consent, the patient was taken for examination. The patient was assessed in a supine position with both anterior superior iliac spine (ASIS) at the same level and the right LL fixed with an Ilizarov ring fixator with three rings (Figure 4). On local examination of the right LL, a wound of 3×2 cm was present over the anterior aspect of the right shin, 10 cm distal to the knee joint. A scar mark from the previous surgery was observed over the lateral aspect of the knee joint. Muscle wasting was seen over the right thigh compared to the left. A flexion deformity of around 20 degrees was seen at the knee joint. Ankle in equinus, great toe in flexion, proximal interphalangeal (PIP) joint in flexion. The bony fragment was observed at the distal 1/3rd of the anterior aspect of the right lower leg. Muscle strength was examined using resisted isometric contraction (RIC) and manual muscle testing (MMT). RIC was weak and painful for the right lower extremity (LE) muscles pre-intervention and strong and painless post-intervention. MMT findings in weeks first, fourth, and eighth are given in Table 2. The range of motion (ROM) was inaccessible for the right LL for some movements pre-intervention due to pain and the presence of an external fixator, however, ROM was assessed in week 4th after the removal of the fixator and in week eight i.e. post-intervention (Table 3). For the right LL, the lower extremity functional scale (LEFS) was taken in weeks one, four, and eigh (Table 4).

**Table 2:** MMT of the right lower extremity in weeks one, four, and eight manual muscle testing: MMT

MMT	Week 1	Week 4	Week 8
Hip			
Flexors	3/5	4/5	4+/5
Extensors	2/5	3+/5	4+/5
Adductors	2/5	3+/5	4+/5
Abductors	2/5	3+/5	4+/5
Medial rotators	2/5	3/5	4/5
Lateral rotators	1/5	3/5	4/5
Knee			
Flexors	1/5	3/5	4/5
Extensors	1/5	3/5	4/5
Ankle			
Plantarflexors	1/5	3/5	4/5
Dorsiflexors	2/5	3/5	4+/5
Invertors	2/5	3/5	4/5
Evertors	2/5	3/5	4/5

**Table 3:** Range of motion of the right lower extremity in weeks one, four, and eight range of motion: ROM, active range of motion: AROM, passive range of motion: PROM, not assessable: NA,

ROM	Week 1		Week 4		Week 8	
	AROM	PROM	AROM	PROM	AROM	PROM
Hip						
Flexion	70˚	75˚	90˚	100˚	100˚	115˚
Extension	15˚	15˚	20˚	25˚	30˚	30˚
Adduction	20-0˚	25-0˚	30-0˚	40-0˚	40-0˚	50-0˚
Abduction	20˚	25˚	30˚	40˚	40˚	50˚
Medial rotation	NA	NA	30˚	35˚	40˚	45˚
Lateral rotation	NA	NA	30˚	40˚	45˚	55˚
Knee						
Flexion	Restricted	Restricted	50˚	60˚	80˚	90˚
Extension	20˚	20˚	50-35˚	60-50˚	80-75˚	90-85˚
Ankle						
Plantarflexion	NA	NA	15˚	20˚	30˚	45˚
Dorsiflexion	NA	NA	20˚	30˚	40˚	40˚
Inversion	Restricted	Restricted	15˚	20˚	30˚	35˚
Eversion	Restricted	Restricted	15˚	20˚	30˚	30˚

**Table 4:** Lower extremity functional scale for the right lower extremity in weeks one, four, and eight lower extremity functional scale: LEFS Scale points- 0: extreme difficulty or unable to perform an activity, 1: quite a bit of difficulty, 2: moderate difficulty, 3: a little bit of difficulty, and 4: no difficultly

Sr. No	LEFS			
	Activities	Week 1	Week 4	Week 8
1.	Any of your usual work, housework or school activities	1	2	4
2.	Your usual hobbies, recreational or sporting activities	0	2	4
3.	Getting into or out of the bath	1	2	4
4.	Walking between rooms	1	2	4
5.	Putting on your shoes or socks	1	2	4
6.	Squatting	0	1	3
7.	Lifting an object, like a bag of groceries from the floor	1	2	3
8.	Performing light activities around your home	2	3	4
9.	Performing heavy activities around your home	1	2	3
10.	Getting into or out of a car	1	2	4
11.	Walking 2 blocks	1	2	3
12.	Walking a mile	0	1	3
13.	Going up or down 10 stairs (about 1 flight of stairs)	0	1	3
14.	Standing for 1 hour	0	1	3
15.	Sitting for 1 hour	1	2	4
16.	Running on even ground	0	0	2
17.	Running on uneven ground	0	0	2
18.	Making sharp turns while running fast	0	0	2
19.	Hopping	0	1	3
20.	Rolling over in bed	1	2	4
	Total score (out of 80)	12/80	30/80	66/80

Therapeutic intervention

The short-term goals were to avoid pulmonary complications, minimize discomfort and swelling, retain and improve the ROM and strength of the joint, deter pressure sores, facilitate walking (PWB) and allow the patient to independently perform activities of daily living (ADLs). The long-term goals were to encourage independent walking with walkers with maximum weight-bearing and limited assistance for everyday activities. Elevation with a pillow was done for positioning the right LL. The physiotherapy rehabilitation protocol was formed to maintain muscle integrity for the right LL and increase the strength of the left LL and both UL to promote independent full weight-bearing walking with a walker and minimal assistance for ADLs. Treatment with rationale is shown in Table 5 and phase-wise treatment protocol is shown in Table 6.

**Table 5:** Therapeutic interventions with rationale active cycle of breathing technique: ACBT, bilateral: B/l, ankle toe movements: ATM, range of motion: ROM, upper extremity: UE, lower extremity: LE, unilateral: U/l, straight leg raise: SLR, dynamic quadriceps: Dynamic Quads, flexion deformity: FD The reference for the above table is Table 4 from Reference No- [12]

Therapeutic interventions	Rationale
Patient education	To prevent anxiety and to ensure adherence to the prescribed physiotherapy protocol.
ACBT and deep breathing exercises	To avoid pulmonary complications.
B/l ATM	To maintain proper blood circulation in the lower limb
Heel slides B/l	To retain quadriceps and hamstring strength, decrease joint stiffness, and increase range.
ROM exercises for B/l UE and LE	To restore ROM to normal or at least the functional range as well as to maintain the available range
Isometrics Exercise	To initiate muscle contraction.
Strengthening of B/l UE with weight cuff	To ensure adequate strength of UE for gait training
Log rolling towards the unaffected side	To prevent pressure sores and to facilitate in-bed mobility
U/l and/or B/l pelvic bridging	To strengthen the back and hip extensors
SLR B/l	To maintain the available range of hip flexion and knee extension and strengthen quadriceps
Dynamic Quads B/l	To maintain the available range of knee extension and strengthen quadriceps
Passive stretches for right side hamstrings and quadriceps	To reduce right knee FD
Bedside sitting	To maintain sitting balance
Wheelchair mobilization	To aid early gait training and make patients independent
Sit to stand with a walker	To improve knee mobility and strengthen quadriceps and glutei
Ambulation with walker	To reinforce the normal gait style and initiate ambulation.
Gait training exercises	To facilitate normal gait and co-ordination and to promote strength

**Table 6:** Phase-wise rehabilitation protocol active cycle of breathing technique: ACBT, BD: twice a day, TD: thrice a day, bilateral: B/l, ankle toe movements: ATM, repetitions: Reps, seconds: secs, active assisted range of motion: AAROM, active range of motion: AROM, lower extremity: LE, straight leg raise: SLR, upper extremity: UE, unilateral: U/l, straight leg raise: SLR, dynamic quadriceps: Dynamic Quads, vastus medialis oblique: VMO, non-weight bearing: NWB, toe touch weight bearing: TTWB, partial weight bearing: PWB, full weight bearing: FWB Reference No [12]

Treatment	Week wise dosage			
	Phase 1 (Inpatient-Day 1 to week 2)	Phase 2 (Inpatient-Week 2 to 4)	Phase 3 (Outpatient-Week 4 to 6)	Phase 4 (Outpatient-Week 6 to 8)
Patient education: The patient and relatives were educated about her current condition, the importance of following the prescribed exercise protocols, the importance of exercise adherence, and how to practice the given exercise protocol, including their repetitions, sets, and durations, with detailed descriptions and illustrations.	Patient education was constantly given in all 4 phases			
ACBT and deep breathing exercises	10 Reps ×10 secs hold ×1 set × BD-TD	10 Reps × 10 secs hold × 2 sets × BD-TD	-	-
B/l ATM	10 Reps × 1 set × BD-TD	10 Reps × 2 sets × BD-TD	10 Reps × 2 sets × BD-TD	10 Reps × 2 sets × BD-TD
Heel slides B/l	10 Reps ×1 set × BD-TD	10 Reps × 2 sets × BD-TD	10 Reps × 2 sets × BD-TD	10 Reps × 2 sets × BD-TD
AAROM exercises for affected LE (Hip Abduction and Adduction)	10 Reps ×1 set × BD-TD	10 Reps × 2 sets × BD-TD	AROM exercises for affected LE (Hip Abduction and Adduction) from week 4 onwards-10 Reps × 1 set × BD-TD	10 Reps × 2 sets × BD-TD
Isometrics B/l for glutei, hamstring, and quadriceps muscle	10 Reps × 10 secs hold × 2 sets × BD-TD	10 Reps × 15 secs hold × 2 sets × BD-TD	-	-
SLR B/l	10 Reps × 1 set × BD-TD	10 Reps × 10 secs hold × 1 set × BD-TD	SLR B/l with 0.5 kg weight cuff -10 Reps × 2 sets × BD-TD	Weight cuff of 1 kg-10 Reps × 2 set × BD-TD
Passive stretches for right side hamstrings and quadriceps	10 Reps × 10 secs hold × BD-TD	5 Reps × 20 secs hold × BD-TD	3 Reps × 30 secs hold × BD-TD	3 Reps × 30 secs hold × BD-TD
AROM exercises with a wand for B/l UE	10 Reps × 1 set × BD-TD	10 Reps × 2 sets × BD-TD	-	-
Log rolling towards the unaffected side	3 Reps × 10 secs hold × BD-TD	5 Reps × 10 secs hold × BD-TD	-	-
B/l LE strengthening with weight cuff	-	Weight cuff of 0.5 kg-10 Reps × 2 sets × BD-TD	Weight cuff of 1 kg-10 Reps × 2 sets × BD-TD	Weight cuff of 1.5 kg-10 Reps × 2 sets × BD-TD
B/l UE strengthening with weight cuff	-	Weight cuff of 0.5 kg-10 Reps × 2 sets × BD-TD	Weight cuff of 1 kg-10 Reps × 2 sets × BD-TD	Weight cuff of 1.5 kg-10 Reps × 2 sets × BD-TD
Pelvic bridging	U/l pelvic bridging 10 Reps × 5 secs hold × 1 set × BD-TD	10 Reps × 10 secs hold × 1 set × BD-TD	B/l pelvic bridging from week 4 onwards- 10 Reps × 5 secs hold × 1 set × BD-TD	10 Reps × 10 secs hold × 1 set × BD-TD
Dynamic Quads B/l	10 Reps × 10 secs hold × 1 set × BD-TD	Dynamic Quads B/l with 0.5 kg weight cuff- 10 Reps × 2 sets × BD-TD	Weight cuff of 1 kg-10 Reps × 2 sets × BD-TD	Weight cuff of 1.5 kg-10 Reps × 2 sets × BD-TD
VMO strengthening B/l in a high sitting position	10 Reps × 10 secs hold × 1 set × BD-TD	VMO strengthening B/l with 0.5 kg weight cuff- 10 Reps × 2 sets × BD-TD	Weight cuff of 1 kg-10 Reps × 2 sets × BD-TD	Weight cuff of 1.5 kg-10 Reps × 2 sets × BD-TD
Bedside sitting	5 minutes	10 minutes	-	-
Sit to stand with a walker	5 Reps × 10 secs hold	10 Reps × 10 secs hold	5 Reps × 15 secs hold	10 Reps × 10 secs hold
Wheelchair mobilization	-	10 minutes × BD-TD	10 minutes × BD-TD	-
Ambulation with walker	NWB on right LE	TTWB on right LE	PWB on right LE	FWB on right LE
Prone and Side SLR	-	-	10 Reps × 1 set × BD-TD	10 Reps × 10 secs hold × 1 set × BD-TD
Gait training exercises: Spot marching, partial wall squats, stepping (forward, backward, and side), stride walking, retro walking, tandem walking, high step walking, and stair climbing	-	-	10 Reps × 1 set × BD-TD for all	10 Reps × 2 sets × BD-TD for all

## Discussion

COM is a common disease among children belonging to the rural population, especially in India [13]. Chronic extremity-related osteomyelitis continues to be a significant cause of sickness and functional limitations in children in India as well as in other developing countries [14]. Trauma, open fractures, surgery, malnutrition, sickle cell disease, low blood pressure, peripheral vascular disease, long-term use of steroids, malignancy, smoking, alcoholism, systemic or local immunocompromise, the presence of a foreign body, and nowadays, the presence of implants like plates and screws, low socioeconomic status, etc. are the predisposing factors for COM [6,7,15-17]. It is usually associated with sickle cell disease. However, in our case report, the patient had no co-morbidity. In our case report, the patient reported a history of falls and had undergone two surgeries previously, and there is the presence of a ring fixator (implant), which could be the reason for COM [4,14]. The incidence of osteomyelitis in the pediatric population was found to be 2.9 per 100,000 children [18]. One of the predisposing factors may be the age of the patient (14 years), as the highest prevalence was observed in children between the ages of five and 15 [19]. The most frequently affected bone is the tibia, which may be due to its poor vascularization and because it is not fully covered by muscles [1,4,13,20,21]. Staphylococcus aureus is the most commonly found infectious organism in the majority of cases [4,16,20]. However, in our case study, the bacterial culture was found to be negative, which is not so common [4,9].

The management of COM must involve a multidisciplinary team consisting of an orthopedic surgeon, radiologist, pathologist, microbiologist, nutritionist microvascular surgeon, psychologist, and skilled orthopedic physiotherapist [3][14]. Physiotherapy helps hasten recovery by improving ambulation and functional independence; hence, it must be involved in the management of COM. The presence of pain badly affected the movements of the patient’s right lower limb and ADLs. The analgesic administration played a significant role in reducing pain. The patient experienced difficulty walking, so our main objective was to encourage independent FWB walking with a walker and ADLs with the least amount of support possible. Prolonged treatment in the case of COM affects ambulation, functional mobility, and independence, which was also the case in our patient [14]. But in our case study, we initiated the physiotherapy management of the patient, which aided in managing the above-mentioned complications and played an important role in making the patient functionally independent. In the majority of the case studies, physiotherapy was excluded in the multidisciplinary approach to the management of COM and so recovery took a long time [3,14,21]. Several complications are associated with the use of the Ilizarov fixator, which includes muscle contractures, joint stiffness, muscle weakness, and joint subluxation [22]. In our case study, the goals of the rehabilitation were formed considering the presence of an Ilizarov ring fixator, which was not seen in previous studies. So we succeeded in improving her ADLs and supervised full-weight-bearing walking. This case study is intended to highlight the significance of the involvement of physiotherapy in improving functional mobility and ambulation in patients with COM treated with sequestrectomy and an Ilizarov ring fixator. Although it is a known and evident fact that physiotherapy is an integral part of the multidisciplinary team managing such cases, a comprehensive week-wise rehabilitation approach has not been provided in any of the previous studies. By considering this need, an integrated week-wise rehabilitation protocol was designed and implemented in the management of chronic tibial osteomyelitis. In addition, it will provide future guidance to other therapists in providing rehabilitation to such patients and help them return to their pre-injury state, and provide a safe return to and independence in ADLs. 

## Conclusions

Chronic osteomyelitis is one of the most common diseases seen among children in the rural population, especially in developing countries like India. It can be treated surgically and with proper rehabilitation to prevent secondary complications. In the present case, during eight weeks of rehabilitation, there was great improvement-noticed stepwise, with alleviation of pain, improvement in range of motion, muscle strength, and gait; and prevention of secondary impairments, which ultimately led to a safe return to daily activities. This case study lays out a comprehensive physiotherapy rehabilitation plan for patients with chronic osteomyelitis treated with sequestrectomy and an Ilizarov ring fixator. The above case study concludes that a multidisciplinary team involving a definite surgical approach and a tailor-made physiotherapy rehabilitation resulted in improved functional independence and ambulation of the patient, which plays a major role in a fast and successful recovery. 
